# In Vitro Evaluation of Essential Mechanical Properties and Cell Behaviors of a Novel Polylactic-*co*-Glycolic Acid (PLGA)-Based Tubular Scaffold for Small-Diameter Vascular Tissue Engineering

**DOI:** 10.3390/polym9080318

**Published:** 2017-07-30

**Authors:** Nuoxin Wang, Wenfu Zheng, Shiyu Cheng, Wei Zhang, Shaoqin Liu, Xingyu Jiang

**Affiliations:** 1 School of Life Science and Technology, Harbin Institute of Technology, 2 Yikuang Road, Nangang District, Harbin 150001, China; wangnx@nanoctr.cn; 2 Beijing Engineering Research Center for BioNanotechnology & CAS Key Laboratory for Biological Effects of Nanomaterials and Nanosafety, CAS Center for Excellence in Nanoscience, National Center for NanoScience and Technology, 11 Beiyitiao, Zhongguancun, Haidian District, Beijing 100190, China; zhengwf@nanoctr.cn (W.Z.); chengsy@nanoctr.cn (S.C.); 3 The University of Chinese Academy of Sciences, 19 A Yuquan Road, Shijingshan District, Beijing 100049, China

**Keywords:** mechanical property, cell behavior, small diameter vascular graft, fibrin glue, rolling

## Abstract

In this paper, we investigate essential mechanical properties and cell behaviors of the scaffolds fabricated by rolling polylactic-*co*-glycolic acid (PLGA) electrospinning (ES) films for small-diameter vascular grafts (inner diameter < 6 mm). The newly developed strategy can be used to fabricate small diameter vascular grafts with or without pre-seeded cells, which are two main branches for small diameter vascular engineering. We demonstrate that the mechanical properties of our rolling-based scaffolds can be tuned flexibly by the number of layers. For cell-free scaffolds, with the increase of layer number, burst pressure and suture retention increase, elastic tensile modulus maintains unchanged statistically, but compliance and liquid leakage decrease. For cell-containing scaffolds, seeding cells will significantly decrease the liquid leakage, but there are no statistical differences for other mechanical properties; moreover, cells live and proliferate well in the scaffold after a 6-day culture.

## 1. Introduction

Cardiovascular diseases have become one of the leading threats to human lives at present [1]. In clinics, great success on large diameter vascular grafts has been achieved by using synthetic polymers (e.g., expanded polytetrafluoroethylene (ePTFE)) as the substitute material [2,3]. However, for small diameter grafts, these materials have suboptimal performance [2,3,4]. In the fields of tissue engineering and regenerative medicine, small diameter vascular grafts have posed a central challenge, which has attracted researchers’ considerable attention. Researchers now find that biodegradable engineered scaffolds, either seeded with cells or not, may be a practical way to address this problem [5,6,7,8,9]. Scaffolds made of material/cell hybrids or material-only will be remodeled into tissue-like structures as the materials degrade and cells infiltrate. These studies have greatly advanced the development of this field and provided us various solutions or tools for small-diameter vascular regeneration.

In 2012, our group developed a stress-induced self-rolling technique to fabricate multi-layered tubular scaffolds by rolling polydimethylsiloxane (PDMS) membranes into three-dimensional (3D) tubes [10]. This method might be a promising solution to small-diameter vascular engineering. However, PDMS is a bio-inert material that cannot degrade in vivo, and thus not an ideal material for blood vessel substitute [11]. Based on this work, recently, our group developed a novel strategy to construct a kind of rolling-based fully biodegradable scaffolds (i.e., polylactic-*co*-glycolic acid (PLGA) in this study) by a single step whose layers are bonded by fibrin glue [12]. This design synthesizes the advantages of several previous strategies. It can realize fabrication of cell-free scaffolds within 10 min and cell-laden scaffolds within 70 min manually with minimal ancillary equipment. Multiple parameters of the scaffolds, such as diameter, wall thickness, mechanical strength, and cell type and distribution in each layer, can be facilely modulated. 

**Figure 1:**
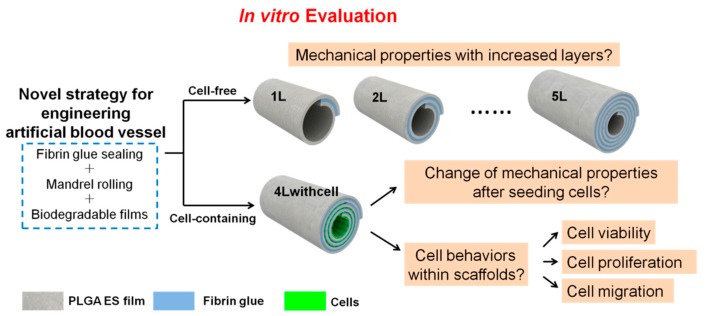
Schematic of in vitro evaluation of the novel artificial blood vessel. Cell-free and cell-containing scaffolds can be fabricated by this method. The substrate material is polylactic-*co*-glycolic acid (PLGA) electrospinning (ES) film. The model cell used is C2C12 mouse myoblast cell. The cell-free scaffolds containing 1.25, 2.25, 3.25, 4.25, and 5.25 layers are noted as 1L, 2L, 3L, 4L, and 5L, respectively. The 4L scaffolds containing cells in its innermost three layers are noted as 4Lwithcell. To fabricate the layered scaffolds without cells, the PLGA films were cut into rectangles with proper sizes, coated with two components of the fibrin glue on two sides of the film, and rolled around an ePTFE mandrel with proper outer diameters by hand. When rolling, the two components of the fibrin glue would react with each other and bond the layers. After the mandrel was gently extracted, the residual glue components in the scaffolds was washed with PBS and eventually only the reacted components that formed fibrin glue would be left in the scaffold. This process is illustrated in Supplementary Materials Figure S2. To fabricate the layered scaffolds with cells, cells were patterned by polydimethylsiloxane (PDMS) chambers. After cell attachment, the chambers were peeled off and the scaffolds were rolled up just the same as those without cells. This process is illustrated in Supplementary Materials Figure S3. In this paper, for cell-free scaffolds, the mechanical property changes with increased layers will be evaluated; for 4Lwithcell scaffolds, the change of mechanical property after seeding cells compared with 4L scaffolds and cell behaviors (cell viability, cell proliferation, and cell migration) within scaffolds will be evaluated.

As the scaffolds would be used as blood vessel substitutes, we carry out several essential mechanical property tests and cell behavior assessments before animal implantation trials (Figure 1). For cell-free scaffolds, evaluation of the mechanical properties (including burst pressure, suture retention, compliance, and so forth) is a critical step to prove their feasibility for implantation; while for cell-laden scaffolds, besides its mechanical properties, cell behaviors (including cell viability, proliferation, and migration) are other vital aspects that should be considered. In this paper, we will report the tests of the mechanical properties of both scaffolds and the results of cell behaviors of cell-containing scaffolds under static culture condition. Our data demonstrate that the mechanical properties of the scaffolds can be modulated by the number of layers and cells survive and proliferate well in the cell-containing scaffolds. These results will guide future application of these scaffolds in animal trials.

## 2. Materials and Methods

### 2.1. Materials

PLGA75:25 (polylactic-*co*-glycolic acid, mass ratio of polylactic acid (PLA) and polyglycolic acid (PGA) is 75:25) polymers (pharmaceutical grade), fibronectin (FN), and the fibrin medical adhesive (Porcine Fibrin Sealant Kit), were purchased from Lakeshore Biomaterials Co., Ltd. (Eden Presley, MN, USA), Sigma Co., Ltd. (Shanghai, China), and Puji Medical Technology Development Co., Ltd. (Hangzhou, China), respectively. The fibrin medical adhesive contains 0.04 mg/mL fibrinectin in its component A, and 450 IU/mL thrombin in its component B. In the fabrication, the two components were both applied 10 μL/cm^2^ on the films. Methyl cellulose solution (1.8%, average molecular weight ranging from 10,000 Da to 220,000 Da) was purchased from BioRoYee Co., Ltd. (Beijing, China). We diluted it into a concentration of 0.25% using distilled water when used for plasma mimics in liquid leakage tests and burst pressure tests according to a published report [13]. Other reagents were all of analytical grade bought from Beijing Chemical Factory. 

### 2.2. PLGA ES Film Preparation

For PLGA electrospinning, PLGA particles were dissolved into the blend of ethyl acetate and dimethyl formamide (DMF) with a mass ratio of 4:1 at 30 (*w*/*w*) %. The temperature and humidity for electrospinning were ~20 °C and ~30%, respectively. The voltage of 12 kV was generated by a direct-current (DC) high-voltage generator (SL150, Spellman, New York, NY, USA). The collection distance was 15 cm and the collection time was around 40 min. All films to be seeded with cells were sterilized by a cobalt ray radiation of 10 kGy, incubated with fibronectin (FN) at a concentration of 20 μg/mL in phosphate buffer solution (PBS) at 37 °C for 1 h to enhance cell adhesion, and washed with PBS once before use.

### 2.3. PMMA Substrate and PDMS Chamber Fabrication

Polymethyl methacrylate (PMMA) substrates with pre-designed patterns were prepared by digitally controlled micromachining. PDMS pre-polymer (base) and catalyst (curing agent) were mixed at a ratio of 10:1, cured against the patterned PMMA substrate, and then incubated at 80 °C for 2 h for sodification. PDMS chambers were then gently peeled off the substrate surface. The fabrication process of PDMS chambers from PMMA substrates was shown in Supplementary Materials Figure S1.

### 2.4. Cell Culture, Staining and Seeding

C2C12 mouse myoblast cells (ATCC, Manassas, VA, USA) were cultured in Dulbecco’s modified Eagle medium (DMEM, Invitrogen, Carlsbad, CA, USA) containing 10% fetal bovine serum (FBS, Invitrogen), 1% penicillin-streptomycin (PS, Invitrogen), 1% Gluta-max (Invitrogen), at 37 °C with 5% CO_2_. Before seeding into PDMS chambers, cells were stained with dyes or not according to different experiments (for cell adhesion, cell viability, and cell proliferation tests, as well as cell migration observed by scanning electron microscope (SEM), the cells were not stained; for cell migration tests using a fluorescent assay, the cells were prelabeled by CellTracker dyes from Invitrogen Co. Ltd). We then collected cells and delivered them into the PDMS chambers at a density of 2 × 10^4^ cm^−2^. Before injecting the cell suspension into designated channels, PDMS chambers had been placed on PLGA films and sealed by fibrin glue to avoid liquid leakage.

### 2.5. Fabrication of Scaffolds with or without Cells

To fabricate the layered scaffolds without cells, the PLGA films were cut into rectangles with proper sizes, coated with two components of the fibrin glue on two sides of the film, and rolled around an ePTFE mandrel with proper outer diameters (in this experiment: 2 mm outer diameter of the mandrel, corresponding a scaffold with 2-mm inner diameter) by hand at a speed of 2–4 cm·min^−1^. When rolling, the two components of the fibrin glue would react with each other to form a sticky fibrin gel and bond the layers. The mandrel was gently extracted and the scaffold was ready. The residual glue components in the scaffolds were washed with PBS and eventually only the reacted components that formed fibrin glue would be left in the scaffold. To investigate the mechanical properties of the scaffolds with different layers, we fabricated the scaffolds without cells containing 1.25, 2.25, 3.25, 4.25, and 5.25 layers (noted as 1L, 2L, 3L, 4L, and 5L, respectively). The extra 0.25 layer was used to anchor the scaffolds and to facilitate the fabrication. This fabrication process is illustrated in Supplementary Materials Figure S2 (using 4L scaffolds as an example). To fabricate the layered scaffolds with cells, cells were seeded on the film with the aid of PDMS chambers. PDMS chambers were used to ensure the cells in the right position and density. The PDMS chamber was peeled off and the scaffolds were rolled up just the same as those without cells. In this experiment, we selected 4 layered scaffolds (4.25 layers) to seeded its innermost three layers with C2C12 cells (noted as 4Lwithcell) to investigate the mechanical properties differences from its 4L alternatives without cells, its cell viability and cell proliferation in each layer, and cell migration between layers. This fabrication process is illustrated in Supplementary Materials Figure S3 (using 4Lwithcell scaffolds as an example).

### 2.6. Measurement of Mechanical Properties

Mechanical properties were measured according to the following protocols. Both cell-free and cell-containing scaffolds were immersed in the culture medium or PBS after fabrication for 20 min at room temperature before measurements were conducted. All tests were applied on three grafts. The room temperature was around 20 °C for all tests.

Suture retention test: one end of a 1.5-cm long graft was clamped at onto a dynamic mechanical analysis machine (DMA, Q800, TA instrument, New Castle, DE, USA). A single bite suture (9-0 Niklon suture, Jiaxin, China) was placed 2 mm from the edge of the other end. A constant pulling rate of 1 mm/min was applied until the suture was pulled out. The maximum force of pulling was recorded as the suture retention.

Burst pressure test: one end of a 2-cm long graft was hermetically clamped, and the other end of the graft was hermetically connected to a syringe on a syringe pump (PHD ULTRA, Harvard, Boston, MA, USA). A constant rate of 50 mL/min was applied to fill 0.25% methyl cellulose solution (plasma mimics) into the graft. The peak pressure before the graft bursts was tested by a pressure gage (AZ 82100 and AZ 8205, Taiwan, China) as the burst pressure. The home-made setup used for burst pressure tests is shown in Supplementary Materials Figure S4.

Wall thickness test: the graft was pressed tightly together and the total thickness of the graft wall was measured by a digital meter. The wall thickness equals the half of the total thickness.

Liquid leakage test: a 2-cm long graft was placed on two vascular catheters, and hermetically sealed with two elastic threads. The graft was flushed with water or 0.25% methyl cellulose solution (plasma mimics) at a pressure of 16 kPa (corresponding to the average systolic pressure of 120 mmHg) for 3 min. The liquid that leaked through the grafts was collected and results were expressed in mL·min^−1^·cm^−2^. The home-made setup used for liquid leakage tests and the following compliance tests is shown in Supplementary Materials Figure S5.

Compliance test: compliance (C) was calculated according to the following equation [14].

(1) $$C=\frac{\frac{D_{\mathrm{inner}}(P2)-D_{\mathrm{inner}}(P1)}{D_{\mathrm{inner}}(P1)}}{P2-P1}\times 10^{4}$$

where P1 and P2 are the lower (80 mmHg) and higher (120 mmHg) pressures, respectively.

(2) $$D_{\mathrm{inner}}=\sqrt{D_{\mathrm{outer}}^{2}-\frac{4(A_{\mathrm{wall}})}{\pi }}$$

where D_inner_ is inner diameter of the graft, D_outer_ is outer diameter of the graft, and A_wall_ is axial cross-sectional area of the vessel wall [15]. A_wall_ is calculated from the cross-sectional area A_wall,0_ at zero pressure [16].

(3) $$A_{\mathrm{wall}}=A_{\mathrm{wall},0}=\pi \cdot h_{0}\cdot \left(D_{\mathrm{outer},0}-h_{0}\right)$$

where h_0_ is wall thickness measured at zero lumenal pressure, D_outer,0_ represents outer diameter measured at zero lumenal pressure. To simplify the calculation, we employed average value of wall thickness for each type of graft as the value of h_0_. We recorded the outer diameter under different lumenal pressures use a digital camera and analyzed using ImageJ 1.43m (NIH USA, 2008) and PhotoShop 6.0 (Adobe).

Tensile elastic modulus:

Tensile elastic modulus was calculated according to the following equations [15]:

Circumferential Stretch Ratio (*λ_θθ_*):

(4) $$\lambda_{\theta \theta }=\frac{D_{\mathrm{inner}}}{D_{\mathrm{inner},0}}$$

Circumferential Ring Strain (*T_θθ_*):

(5) $$T_{\theta \theta }=\frac{1}{2}(\lambda_{\theta \theta }^{2}-1)$$

Cauchy Stress (*σ_θθ_*):

(6) $$\sigma_{\theta \theta }=\frac{P\cdot D_{\mathrm{outer}}\cdot \lambda_{\theta \theta }}{2h_{0}}-P$$

We calculated tensile elastic modulus (*E*) within the physiologic pressure range from the slope of stress-strain curves between 80 mm Hg and 120 mm Hg lumenal pressure:

(7) $$E=\frac{\sigma_{\theta \theta }(P120)-\sigma_{\theta \theta }(P80)}{T_{\theta \theta }(P120)-T_{\theta \theta }(P80)}$$

### 2.7. Cell Viability Test

C2C12 cells (without staining) were seeded on the films with the aid of PDMS chambers at the density of 2 × 10^4^ cm^−2^ (as shown in Supplementary Materials Figure S3). After fabrication and culture of the scaffolds in DMEM for 3 or 6 days, the scaffolds were unrolled. The cells in each layer were then washed with PBS and stained with the LIVE/DEAD kit (Invitrogen). The image was observed with confocal microscopy. 

### 2.8. Cell Proliferation Test by a Fluorescent Assay

C2C12 cells (without staining) were seeded on the films with the aid of PDMS chambers at a density of 2 × 10^4^ cm^−2^ (as shown in Supplementary Materials Figure S3). After fabrication and culture of the scaffolds in DMEM for 3 or 6 days, the scaffolds were unrolled. The cells in each layer were washed with PBS and then lysed (cell culture lysis reagent part #E153A, Promega, Madison, WI, USA) using 1 mL 1× Lysis Buffer. The cell density per layer was measured using a cell proliferation assay according to the manufacturer’s protocol (CyQuant Cell Proliferation Assay Kit, Invitrogen). Briefly, 1/10 of the buffer containing lysed cells was collected in a 96-well plate. Add CyQUANT^®^ GR dye (a proprietary dye that exhibits strong fluorescence enhancement when bound to nucleic acids, with a maximal excitation at 480 nm and a maximal emission at 520 nm) into the buffer to reach a final working concentration of 1× and a total volume of 100.5 μL in each well. After incubating at room temperature for 30 min, the 96-well plate was scanned in a microplate reader (EnSpire Multimode Plate Readers, PerkinElmer, Waltham, MA, USA). We used the excitation light at 480 nm, and collected the emission light at 520 nm. Three cell-seeded grafts were used in this test. We also plotted a standard curve of the relationship between the cell number and emission intensity at 520 nm. Briefly, the cells were collected and counted after trypsinization, and then the cell suspensions containing 100,000, 75,000, 50,000, 25,000, 12,500, and 6000 cells were pelleted using a centrifuge at 1200 rpm for 5 min. After discarding the supernatant, 100.5 μL CyQUANT^®^ GR dye-containing cell lysis was added into the collected cells and the solution was transferred into 96-well plates. After incubation at room temperature for 30 min, we read the emission intensity at 520 nm. The background of each sample (the intensity of sample without containing cells) has been deducted. We converted the emission intensity value of tested samples into cell number per square centimeters.

### 2.9. Cell Migration Test by a Fluorescent Assay

Pre-labeled C2C12 cells were seeded onto the films with the aid of PDMS chambers at a density of 2 × 10^4^ cm^−2^ (as shown in Supplementary Materials Figure S6). The cells on the first, second, and third layer were stained with CellTracker Green, CellTracker Orange, and CellTracker DeepRed, respectively. After fabrication and culture of the scaffolds in DMEM for 3 or 6 days, the scaffolds were unrolled. Random fields of each layer were scanned with all three excitation/emission (ex/em) waves: Cell Tracker Green- (ex/em) 488/517 nm, Cell Tracker Orange- (ex/em) 543/565 nm, and Celltracker Deep Red- (ex/em) 633/650 nm. The spectra windows for emission collection were set so that there was no cross-talk among the three dyes. If the stained color in some layers occurs to other layers, it demonstrates that the migration takes place from some layers to other layers.

### 2.10. Cell Adhesion, Proliferation, and Migration Tests by SEM

To investigate the cell adhesion, proliferation, and migration by SEM, we seeded cells without staining only on the second layer of the 4L scaffolds with the aid of PDMS chambers at the density of 2 × 10^4^ cm^−2^ (as shown in Supplementary Materials Figure S7). Cell adhesion was checked after 12 h of incubation in a cell culture incubator. After culture of 3 days and 6 days, we unrolled the scaffolds. Cell proliferation on the second layer of the scaffold was checked by SEM. Cell migration from the second layer to the first, third, and fourth layer was checked by SEM. The flat film and the unrolled scaffolds were fixed in 4% paraformaldehyde for 2 h, washed with distilled water for 3 times, and dehydrated in gradient using 30%, 50%, 70%, 80%, and 90% each once and 100% ethanol for three times. Each dehydration was performed for 5–10 min. The samples were then air dried at room temperature, mounted on a metal stub, and sprayed with gold for 60 s before SEM observations.

### 2.11. Film Thickness, Fiber Size, Pore Size, and Cell Size Measurements

The thickness of the films was measured by an electronic digital micrometer (Guanglu, Guilin, China). The average fiber diameter was measured from SEM images; 200 fibers were manually measured and analyzed using ImageJ software (NIH USA, 2008). From SEM images, pore sizes and cell sizes were measured by manually fitting an ellipse in representative pores formed by fibers in the same plane and cells adhering on the film. The size of each pore or cell was the average between the long and short diameters of the fitted ellipse; 100 pores and 50 cells were manually measured and analyzed using ImageJ software. Results are given as mean ± standard deviation.

### 2.12. PLGA Film Degradation

To test the degradation property of ES PLGA films, the films were immersed into DMEM medium, and incubated at 37 °C with 5% CO_2_ for 2 weeks. The films were washed with distilled water three times, lyophilized using a lyophilizer (FD-1A-50, Biocool, Beijing, China), and observed by SEM.

### 2.13. Statistics

Differences between two groups were examined via unpaired two-tailed Student’s *t*-test using GraphPad Prism 6.0 software (GraphPad Software, Inc., La Jolla, CA, Country). A value of *p* < 0.05 was considered to be statistically significant.

## 3. Results

### 3.1. PLGA ES Film Characterization and Mechanical Properties for Scaffolds without Cells

**Figure 2:**
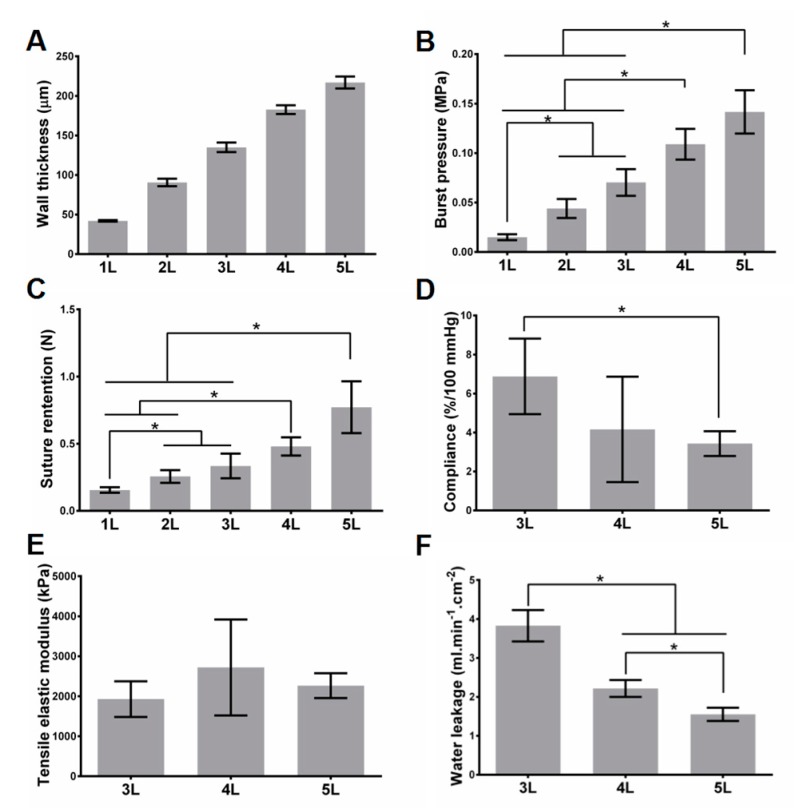
Mechanical properties for cell-free scaffolds. (**A**–**C**) Wall thickness (**A**), burst pressure (**B**), and suture retention (**C**) of scaffolds with 1–5 layers. (**D**–**F**) Compliance (**D**), tensile elastic modulus (**E**), and water leakage (**F**) of scaffolds with 3–5 layers. Data of (**D**,**E**,**F**) do not have the results of 1 and 2 layered scaffolds, because the scaffolds with 1 and 2 layers cannot bear the pressure of 120 mmHg. All tests were biological triplicates. * indicates the *p* value smaller than 0.05.

The PLGA films possess a thickness of 41.3 ± 2.1 μm (*n* = 10). The fiber size is 0.59 ± 0.28 μm (*n* = 200) and the pore size of the ES film is 2.92 ± 1.76 μm (*n* = 100) (Supplementary Materials Figure S8). With the increase of the layer number (1 to 5), the wall thickness increases almost linearly (from 42 ± 1 μm to 200 ± 8 μm) (Figure 2). The burst pressure also increases from 0.015 ± 0.0017 MPa for a 1-layered scaffold to 0.142 ± 0.013 MPa for a 5-layered scaffold. The suture retention increases with layer numbers, from 0.16 ± 0.021 N for 1 layer to 0.77 ± 0.19 N for 5 layers. For the compliance, modulus, and water leakage tests, because scaffolds with 1 and 2 layers cannot bear the pressure of 120 mmHg, we could only obtain the data of these parameters for scaffolds with 3–5 layers. Compliance between 80 mmHg and 120 mmHg decreases with the layer number, from 6.88 ± 1.94%/100 mmHg for 3 layers to 3.41 ± 0.64%/100 mmHg for 5 layers. Under 120 mmHg pressure, we observe a similar decreasing trend on water leakage (3.83 ± 0.23, 2.22 ± 0.13, 1.55 ± 0.10 mL/(min·cm^2^) for 3, 4, 5 layers, respectively and 0.25% methyl cellulose solution leakage (0.85 ± 0.17, 0.55 ± 0.076, 0.23 ± 0.045 mL/(min·cm^2^) for 3, 4, 5 layers, respectively) (Figure 2 and Supplementary Materials Figure S9). Tensile elastic modulus has no significant changes with the increase of layer number, and values are 1929 ± 257, 2719 ± 693, and 2264 ± 181 kPa for 3, 4, and 5 layers, respectively. The pressure-inner radius curves show that the radius of scaffolds with 3–5 layers increases steadily with the increase of lumenal pressure (Supplementary Materials Figure S10). According to Supplementary Materials Table S1, there are no significant differences for circumferential ring strain for 3–5 layered scaffolds at 80 and 120 mmHg, but Cauthy stress significantly decreases from 3–5 layered scaffolds at 80 and 120 mmHg. In addition, the film shows slight degradation and apparent shrinkage after a 2-week incubation (Supplementary Materials Figure S8).

### 3.2. Mechanical Properties for Scaffolds with Cells

**Figure 3:**
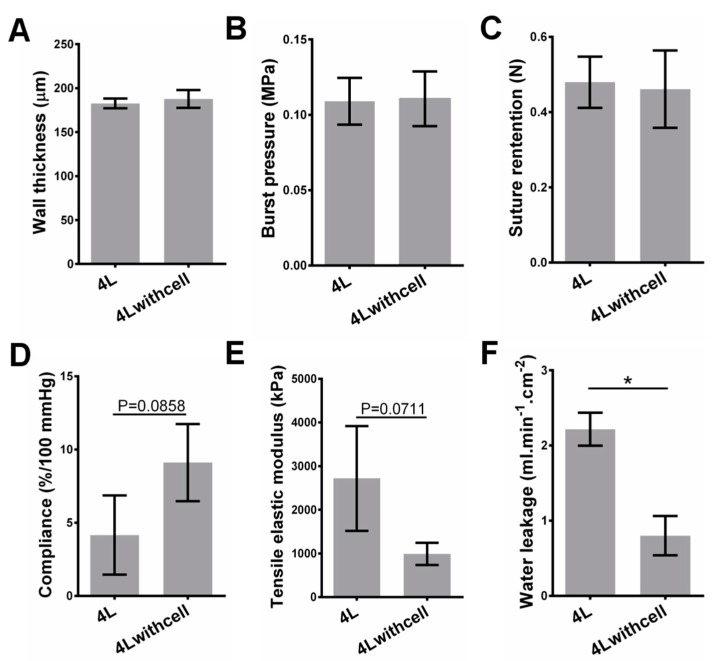
Comparison of mechanical properties of 4 layered scaffolds with and without cells. Wall thickness (**A**), burst pressure (**B**), suture retention (**C**), compliance (**D**), tensile elastic modulus (**E**), and water leakage (**F**). All tests were biological triplicates. * indicates the *p* value smaller than 0.05.

Because the typical structure of a blood vessel includes three cell layers [17], we then compared the mechanical properties of 4 layered scaffolds with and without 3 cell-containing layers (the scaffolds with cells contain cells in their 1st to 3rd layers, and the 4th layer as the outermost layer does not contain cells, in order to reinforce the whole structure). The cell size on the film is 30.5 ± 7.4 μm (*n =* 50), much larger than the pore size (Supplementary Materials Figures S8 and S10). Incorporating cells does not significantly increase the wall thickness, burst pressure, suture retention, compliance, and tensile elastic modulus of the scaffold (Figure 3). The compliance and tensile elastic modulus appear to have significant changes, but there are no statistical significances (*p* = 0.0858 and *p =* 0.0711, respectively). Liquid leakage of scaffolds containing cells has a sharp decrease compared with that of the cell-free ones (0.80 ± 0.15 vs. 2.22 ± 0.13 mL/(min·cm^2^) for water, and 0.17 ± 0.036 vs. 0.55 ± 0.076 mL/(min·cm^2^) for 0.25% methyl cellulose solution) (Figure 3 and Supplementary Materials Figure S9). The pressure-inner radius curves show that the radii of both 4L and 4Lwithcell scaffolds increase steadily with the increase of lumenal pressure (Supplementary Materials Figure S11). There are no significant differences on circumferential ring strain both at 80 and 120 mmHg and Cauthy stress for 4L and 4Lwithcell scaffolds at 120 mmHg. However, Cauthy stress of 4Lwithcell scaffolds has a significant decrease compared with that of 4L (Supplementary Materials Table S1). 

### 3.3. Cell Behaviors in Cell-Containing Scaffolds

After evaluation of the mechanical properties, we tested the cell behaviors in cell-containing scaffolds, including cell viability, cell proliferation, as well as cell migration.

#### 3.3.1. Cell Viability in Each Layer

**Figure 4:**
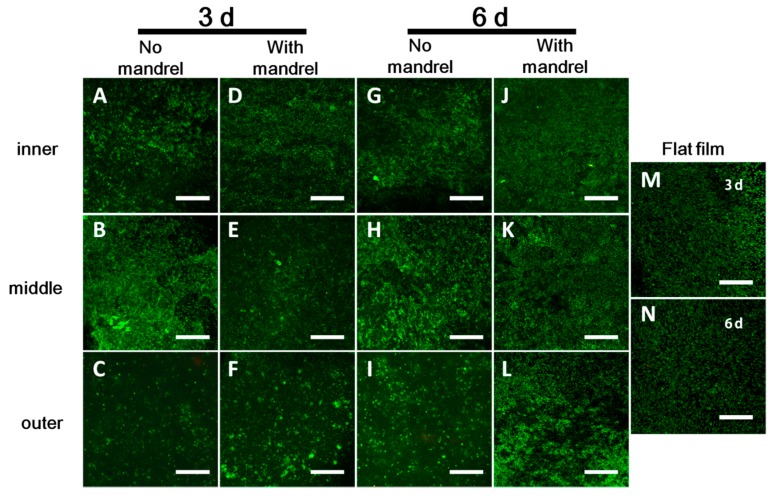
Cell viability at 3 (**A**–**F**) and 6 (**G**–**L**) days with (**D**–**F**,**J**–**L**) and without (**A**–**C**,**G**–**I**) mandrel. (**A**,**D**,**G**,**J**) inner layer. (**B**,**E**,**H**,**K**) middle layer. (**C**,**F**,**I**,**L**) outer layer. (**M**) cell viability at 3-d culture on flat film (**N**) cell viability at 3-d culture on flat film. Green: live cells. Red: dead cells. The model cell used is C2C12 cell. Each image is chosen randomly as the representative image in corresponding layers. Scale bars: 200 μm.

We tested the cell viability of the scaffolds either with or without the mandrel, namely, the ePTFE mandrels were pulled out or not when incubating the scaffolds in the culture medium. After static culture of 3 and 6 days, we stained the cells on the unrolled films in each layer using LIVE/DEAD Kit. From the confocal images, we can rarely see the PI-positive (red, indicating dead cells); almost all the cells are Calcein-positive live cells (green) (Figure 4). There are no obvious differences of the cell viability between the scaffolds with and without mandrel and also between the tested scaffolds and the flat film serving as the control. Moreover, this phenomenon is not affected by culturing time (3 days or 6 days). These results demonstrate that the cells in scaffolds possess high viability.

#### 3.3.2. Cell Proliferation in Each Layer

**Figure 5:**
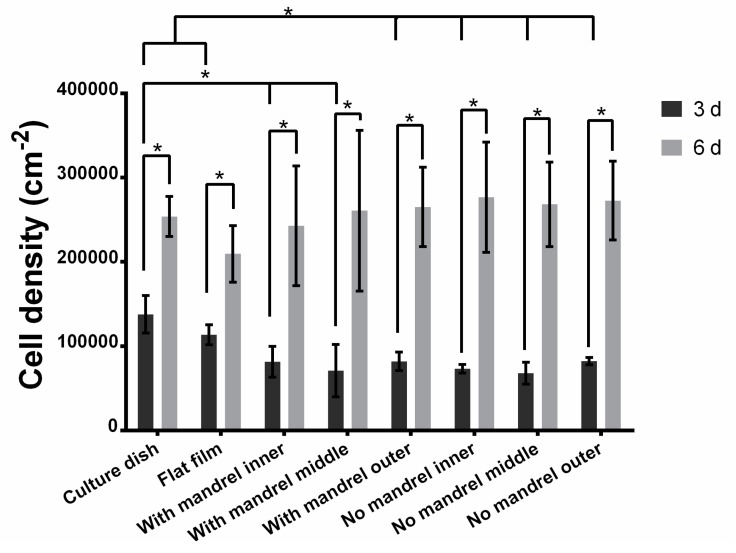
Cell proliferation at 3 and 6 days with or without mandrel. All tests were biological triplicates. * indicates a *p* value smaller than 0.05.

We further tested the cell proliferation in each layer. The standard curve of cell number (*c*) and fluorescent intensity (*f*) shows a well linear relationship as shown in Supplementary Materials Figure S12 (*f* = 0.7885*c* − 2994, R^2^ = 0.9983). Compared with cells cultured in the scaffolds for 3 days, cells cultured for 6 days in each layer have a significant growth, regardless of the scaffolds incubated with or without mandrel (Figure 5). However, it is obvious that cells growing in almost all scaffolds have less cell proliferation than cells growing on culure dishes and flat films after culture of 3 days, while no statistical significance between the cells in scaffolds and those on flat films is observed for cells after culture of 6 days (Figure 5). These results indicate that cells in different scaffolds and layers have a similar proliferation trend, but there are differences on proliferation rate. The cell proliferation in the scaffolds is also proofed by SEM image (Supplementary Materials Figures S10 and S13). With the increase of time, the cell density on the PLGA film or unrolled scaffold film increases.

#### 3.3.3. Cell Migration between Layers

**Figure 6:**
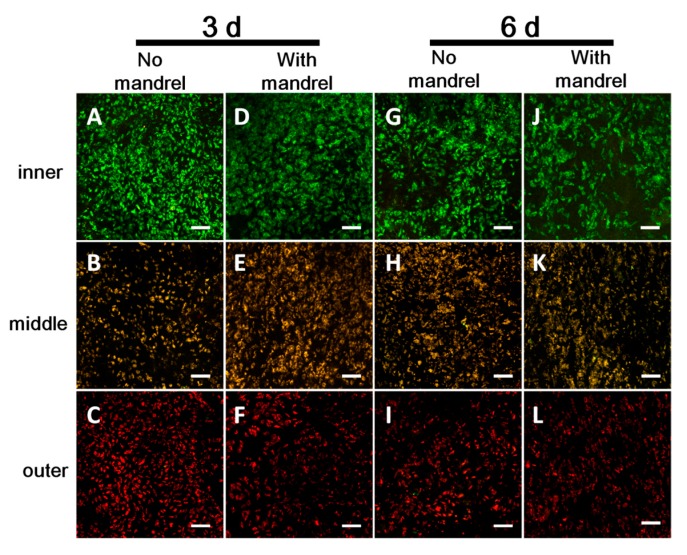
Cell migration at 3 (**A**–**F**) and 6 (**G**–**L**) days with (**D**–**F**,**J**–**L**) and without (**A**–**C**,**G**–**I**) mandrel. (**A**,**D**,**G**,**J**) inner layer. (**B**,**E**,**H**,**K**) middle layer. (**C**,**F**,**I**,**L**) outer layer. The model cell used is C2C12 cell. Each image is chosen randomly as the representative image in corresponding layer. The cells seeded on each layer were pre-labeled with different dyes. After unrolling, each image is chosen randomly as the representative image in corresponding layer. If the migration between layers occurs, the pre-labeled cells will appear on another layer. Scale bars: 50 μm.

Cell migration was assessed by the movement of pre-labeled cells between layers. If the migration occurs, some cells pre-labeled in previous layer will be found in other layers. In our case, no matter for scaffolds with or without mandrel, and no matter for scaffolds of 3 or 6 days culture, there is almost no cell migration between layers (Figure 6). The cell proliferation in the scaffolds is also proofed by SEM image (Supplementary Materials Figure S13). After culture of 3 and 6 days, very rare cells seeded in the second layer of the scaffold migrated to other layers. These results indicate that quite limited migration occurs in the scaffolds, which is largely affected by the pore size in the scaffold [18,19,20,21]. 

## 4. Discussion

Employing the bi-component fibrin biomedical glue, we have developed a rapid and straightforward method to fabricate layered tubular scaffolds by rolling biodegradable polymer thin films around a removable smooth mandrel [12]. In order to overcome the limitations of several reported methods also based on rolling, the following efforts were made: (i) biomedical glue was used to stabilize the whole structure and reduce the layer fusion time [22]; (ii) bioabsorbable electrospinning (ES) polymer films were used to provide an easy-to-fabricate, easy-to-store, extracellular matrix (ECM)-like substrate [23]; (iii) cell suspensions were used to lessen production time for cell sheets [24]. We also inherit or imitate the advantages of the existing methods [10,22,25,26]. For example, rolling-based methods can tune the inner diameter of the tube precisely in virtue of the mandrel; the concept is simple and easy-to-learn without complex manipulations, harsh conditions, and expensive equipment; the well-designed PDMS channel is an effective tool to ensure the accurate cell distribution on the film. A number of published reports have employed rolling-based manufacture of three dimensional (3D) tubular structure from two dimensional (2D) films [24,27,28,29]. It is worth mentioning that, rolling 2D film into 3D structures has the intrinsic advantage that we can modify or pattern the film very conveniently with cells, molecules, and/or nanoparticles because of the booming development of various patterning techniques on 2D substrates, which may favor their future applications [30,31,32,33]. Therefore, besides the rapidness, flexibility and simplicity, simple and controlled modification of cells or other components in the specific location in 3D structures is one of the most important features of our method.

According to the scaffolds encapsulating cells or not, the prepared scaffolds can be classified into two types: cell-free or the cell-containing scaffolds. The two types of scaffolds have different advantages [6]. Generally speaking, the cell-free scaffolds possess the features of the short fabrication time and ease of storage, while cell-containing scaffolds have a more similar structure to real blood vessels and better biocompatibility. Both kinds have been intensively investigated and promising results have been acquired to further the development of the vascular tissue engineering field [34,35,36,37,38]. Herein, we applied the fibrin glue-assisted rolling technique to fabricate both kinds of vascular grafts.

Realizing tunable mechanical strength effectively is one of the features of our scaffolds. For cell-free scaffolds, as expected, the burst pressure and suture retention increase with the increase of the layer number, while water leakage and compliance decrease with the increase of the layer number. Controlling layer number is a straightforward way to tune the mechanical strength, and the fibrin glue ensures the integrity of the stacked layers in our strategy. For cell-containing scaffolds, compared with the cell-free variant with the same layers, the water leakage significantly decreases, but there are no significant changes on other mechanical properties. Although the innermost three layers of the scaffolds contain cells (see Materials and Methods section), it is not difficult to image that the mechanical properties can also be controlled by the number of outer layers.

As a scaffold that can be implanted, the most essential requirements on mechanical strength include that: it can withstand the pressure of blood, can be easily sutured by doctors, and can confine the blood in the scaffold without severe leakage. Thus, burst pressure, suture retention, and leakage property should be the three most important mechanical properties, for either cell-free scaffolds or cell-containing scaffolds. Previous reports have demonstrated that the human saphenous vein, which is regarded as one of the most appropriate substitutes in cardiovascular clinic practice, possesses the burst pressure of larger than 1680 mmHg (0.223 MPa) [22,39,40,41,42]. In the present study, the burst pressure of 5-layered PLGA scaffold approaches this value. Another important merit of this method is the flexibility on the choice of materials. As for the material, PLGA may seem soft compared with some commonly used materials such as poly(*ε*-caprolactone) (PCL) and PLA. Thus, if these stronger materials are used, the layer number reaching the implantable level would be reduced. Suture retention is a basic index that ensures the scaffold can be compatible with the suture in the surgery. The reported suture retention of the human saphenous vein was ~196 gf (~1.92 N) [39,41]. However, there are examples for implantable blood vessel substitutes with much lower suture retention reported, such as the fast degradable and highly elastic poly(glycerol sebacate) (PGS) graft (suture retention: ~0.45 N) [43,44]. This value is not a challenge for our rolling-based strategy. As for the leakage property, our scaffolds have shown that the uncontrolled quick leakage can be avoided, and the value can be further reduced by increasing layer number and/or cell seeding. The cells will cover some pores on the film and thus induce the leakage decrease (Supplementary Materials Figures S8 and S10). The leakage is also greatly reduced using methyl cellulose solution as plasma mimics with a higher viscosity than water. We believe that the leakage will be further reduced when using real blood, because of its higher viscosity than plasma and the existence of the clotting mechanism. It is noted that the leakage should not be completely stopped because the leakage also reflects the free nutrient transportation in the scaffold to some extent. The literatures have documented that cells in scaffolds without pores/zero leakage have low viability [18,19,20].

Other mechanical properties, such as compliance and elastic modulus, are also very important. Compliance mismatch has been regarded as a key factor for vascular stenosis at post-implantation [45,46]. As a result, implantation of a scaffold with compliance and elasticity similar to those of native vessels is the most favorable [47,48,49]. However, whether cell-free or cell-containing scaffolds, they will undergo complex and extensive in vivo evolution, including cell infiltration, scaffold degradation, ECM deposition, and so forth. These progresses will gradually but substantially alter the structure and thus the compliance, elasticity or modulus of the scaffolds. Typically, the compliance of the scaffolds will become increasingly similar to that of the host vessels. The compliance and elasticity are mainly derived from infiltrated smooth muscle cells (SMCs) and biosynthesized elastin. Elastin is secreted by synthetic type of SMCs and fibroblasts (FBs), and thus colonization of SMCs and FBs as well as inducing the transformation of more synthetic SMCs is the pre-requisite of elastin formation. Among multiple types of ECM proteins in the blood vessel wall, elastin takes up to 50% of its dry weight. Because elastin can be stretched under load and recoil to its original configuration when the load is removed, it can determine the elasticity of the blood vessel [16,49,50]. Thus, the design for enhancing elastin formation should be considered in our future exploration. It is also noted to mention that another major ECM protein, collagen, plays a critical role in supporting the whole structure of the graft and strengthening the graft in vivo [17,51]. It possesses a structure of elongated fibrils in blood vessels. It is essential for maintaining and enhancing burst pressure and suture retention of the graft. For cell-containing scaffolds, the cell behavior in vitro can be used to predict the cell behavior in vivo [52]. Interestingly, the cells in our scaffolds live and proliferate well. These observations indicate that the nutrients can be continuously supplied to cells and the wastes can also be discharged promptly. Considering that there are no vessels to transport the nutrients and wastes, the process is free diffusion. It is known that the diffusion limit for the tissues is less than 200 μm. Beyond this value, the cells will not obtain enough nutrients and can undergo apoptosis. This phenomenon has been well documented in many literatures [53,54]. Our cell-containing scaffold contains three layers of cells in its innermost layers, and the third layer of cells has a maximal distance of less than 100 μm towards the lumen of the vessel (estimated from half of the wall thickness data of 4 layered scaffolds with cells shown in Figure 3), within the diffusion limit of 200 μm. This is the reason why the cells live and proliferate well in the scaffold. However, the cell proliferation rate in different layers is different. We speculate that it is because of the different distribution or different transportation ratio of nutrients, oxygen, and wastes as well as the different distribution of cells [18]. Cell migration is an important process for scaffold evolution. The scaffold evolution is a self-assembly process after the scaffolds are implanted into the hosts. In this stage, seeded cells and cells in hosts interact with each other, and are remodeled into an ordered structure that becomes more and more analogous to native tissue with the evolution time. The pore size plays a significant role in cell migration [18,19,55]. In our scaffold, because of the small fiber diameter-induced small pore size, the cell migration between layers has been greatly confined (Figure 6 and Supplementary Materials Figures S10 and S13). We believe that there may be some disadvantages, such as insufficient cell interactions with hosts cells and potential insufficient waste clearance. Thus, in future assays, we will use ES sheets with thicker fibers and thus larger pores to facilitate the tissue evolution.

In addition to the above discussion, there are still some issues to be addressed in our scaffold design or in our future study. First of all, an intrinsic feature of the “rolled sheet” design is a longitudinal internal ridge formed by the edge of the inner layer. The edge may foster the blood cell adhesion and complicate the endothelialization. A possible solution to smoothen this edge may be using a substrate with gradually increased thickness at its one edge (Supplementary Materials Figure S14). Second, our method faces some difficulties when fabricating very long grafts, because it is rather hard to control long films well in a certain direction when rolling manually. We believe a rolling machine can be employed to address this problem, but it will sacrifice some simplicity of our method [56]. Third, although in this study we evaluated several essential mechanical properties for the scaffold, it is far from a detailed complete evaluation. If we want to reach higher ambitions, much more complex mechanical properties should be carried out. One of the examples is that grafts need to be pliable either to lead them over the curved heart surface, or to allow movements if used as a popliteal graft. Another example is that the graft should resist kinking as this would obstruct blood flow. Unfortunately, our manufacturing cannot address these requirements in its current state. We think a graft made of some kind of highly-elastic material, such as PGS, with a wrinkled graft wall may be a potential solution for these two challenges. Fourth, in our study, we used 2 cm long grafts for most tests, and the length is too short for applications in humans. As a blood vessel scaffold in its initial stage, our short term goal is to verify its feasibility in small animal implantation, such as the rabbit. The 2 cm graft is long enough for this implantation. In the next stages of the evaluation of the blood vessel graft, such as large animal implantation and clinical trials, longer grafts should be used. Also, the applications of the graft in humans vary according to the blood vessel replaced in different body regions. These applications require the blood vessel featuring different biomechanical properties. At the current stage, we have not yet considered which specific region our graft will be used for (e.g., coronary artery bypass, renal dialysis, or popliteal graft). Our focus in this stage is characterization of the basic properties of the graft. In the future, we will concentrate on coronary artery bypass in an initial plan. Fifth, in this work, although we evaluated cell behaviors within 6 days using the well-established C2C12 cells based on published reports [18,52,57], longer-term exploration should be carried out. We think in this exploration, the cells used should be blood vessel-related cells or stem cells from real patients. The graft should be incubated in a perfusion system for weeks or even longer. The formation of ECM and acquirement of contractile activity have been reported for blood vessel grafts in similar systems [58]. These provide the artificial blood vessels more analogous structure and function like the native blood vessels; although the formed thrombogenic matrix components such as collagen may be a risk factor if the grafts are exposed to blood. This issue could be addressed by seeding a confluent endothelial layer and introducing signaling factors to recruitment anti-thrombogenic cells (e.g., using CD34 antibody to home endothelial progenitor cells in circulating blood) [59]. The ECM deposition might be modulated beneficially by modifications to the polymer matrix. For example, gold nanoparticles conjugated to Type I collagen can greatly improve the resistance to collagenase of the collagen construct [60]. Sixth, it is worth investigating whether the orientation of the burst point of the scaffold in burst pressure tests depends on the orientation of the nanofibers in the ES mats. Seventh, due to inevitable blood leakage through the graft wall, it is necessary to know how the blood components interact with the polymer matrix. A clear investigation on the mechanisms, e.g., just reducing the permeability, initiating the clotting, or others, is beneficial to design an improved blood vessel graft. Finally, as we noted, the liquid flux from the graft wall probably helps to nourish the cells in the scaffold, but it will be significantly reduced by the presence of cells; therefore, it is important to know where the balance is between the cell density and the amount of liquid flux to sufficient nutriment for cells in the scaffolds.

## 5. Conclusions

In conclusion, we have demonstrated that the mechanical properties of our rolling-based scaffolds can be tuned by the number of layers. This feature makes our scaffolds implantable by changing the layer number according to the selected substrate materials. The scaffolds possess controlled liquid leakage with the aid of fibrin glue and cell seeding will further decrease the leakage. Cells live and proliferate well in the scaffold. These evaluations suggest that our newly designed strategy for vascular graft fabrication, featuring rolling biodegradable films around a mandrel and sealing the layers by fibrin glue, possesses satisfactory mechanical properties if using an appropriate layer number and favorable cell compatibility in vitro, which may be a promising solution to small diameter vascular grafts. In the future, we need to increase the fiber diameter to improve the cell migration between layers. The work in the next stage is an animal implantation test based on these mechanical properties and cell behavior evaluation data.

## Supplementary Materials

The following are available online at www.mdpi.com/2073-4360/9/8/318/s1. Figure S1: Fabrication PDMS chambers from PMMA substrates. Figure S2: Fabrication scaffolds without cells. Figure S3: Fabrication scaffolds with cells. Figure S4: Setup for burst pressure test. Figure S5: Setup for water leakage and compliance tests. Figure S6: Fabrication scaffolds with stained cells (illustrating the cell distribution for cell migration test by a fluorensent assay). Figure S7: Fabrication scaffolds with one layer of cells seeded (illustrating the cell distribution for cell adhesion, proliferation and migration tests by SEM). Figure S8: The morphology of PLGA fibers before and after 14 days’ degradation in DMEM at 37 °C with 5% CO_2_. Figure S9: The 0.25% methyl cellulose solution leakage from the scaffolds. Figure S10: Cell adhesion after 12 h on PLGA ES films. Figure S11: The pressure-inner diameter curve of the scaffolds. Figure S12: The calibrated standard curve of the CyQuant Cell Proliferation Assay Kit. Figure S13: SEM images of and cell proliferation and cell migaration after 3 and 6 days in scaffolds. Figure S14: A possible design for the solution to the internal edge of the rolling-based scaffolds. Table S1: The relationship between circumferential tensile strain and the Cauthy stress of the scaffolds.
