# Supplementary material for: In Vitro Evaluation of Essential Mechanical Properties and Cell Behaviors of a Novel Polylactic-co-Glycolic Acid (PLGA)-Based Tubular Scaffold for Small-Diameter Vascular Tissue Engineering
Source: Polymers (Basel). 2017 Jul 30;9(8):318. doi: 10.3390/polym9080318 (PMC6418786; doi:10.3390/polym9080318)
Supplement: Supplementary file 1 [file polymers-09-00318-s001.pdf]

polymers

MDPI

Supplementary Material

In vitro evaluation of essential mechanical properties and cell behaviors of a novel PLGA-based tubular scaffold for small-diameter vascular tissue engineering

Nuoxin Wang, Wenfu Zheng, Shiyu Cheng, Wei Zhang*, Shaoqin Liu* and Xingyu Jiang*

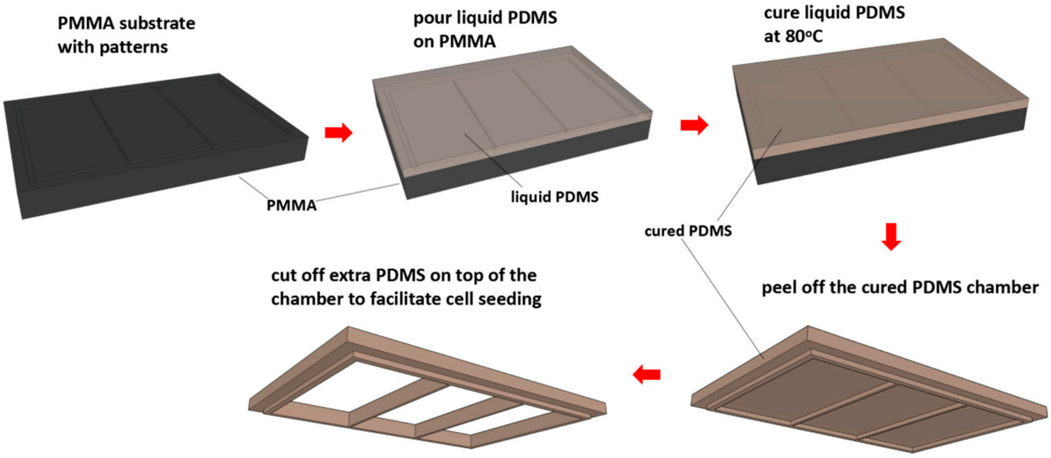

Figure S1. Fabrication PDMS chambers from PMMA substrates. The predesigned patterns were carved on PMMA substrate by digitally controlled micromachining. The liquid PDMS was poured on the surface of the PMMA with patterns. After curing the liquid PDMS at 80°C for 120 min, we peeled off the cured PDMS from the PMMA substrate. The patterns against the PMMA substrate were on the PDMS surface. The extra PDMS on top of the chamber was then cut off to facilitate cell seeding. Please see also ref. 1 for a more detailed instruction of the use of the chamber.

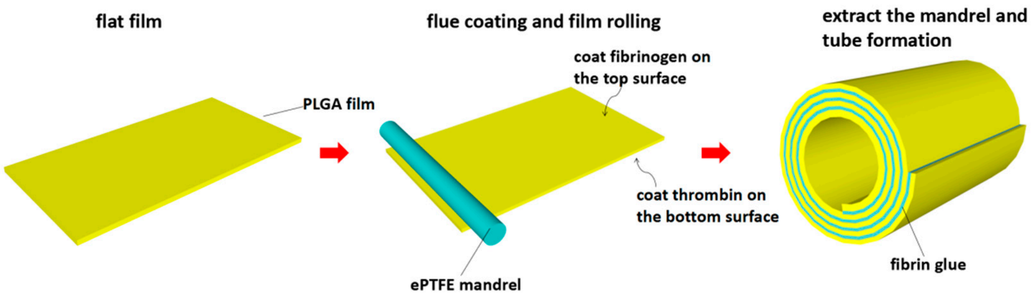

Figure S2. Fabrication scaffolds without cells. The PLGA films were cut into rectangles with proper

sizes, coated with two components of the fibrin glue on two sides of the film, and rolled around an ePTFE mandrel with proper outer diameters by hand. When rolling, the two components of the fibrin glue would react with each other to form a sticky fibrin gel and bond the layers. The mandrel was gently extracted. The residual glue components in the scaffolds was washed with PBS (not shown in the figure) and eventually only the reacted components that formed fibrin glue would be left in the scaffold. Please see also ref. 1 for a more detailed instruction of the fabrication process.

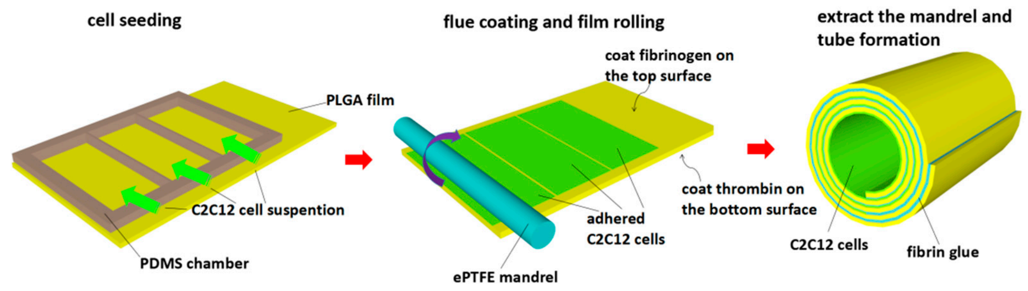

Figure S3. Fabrication scaffolds with cells. The PLGA films were cut into rectangles with proper sizes. Cells were seeded on the film with the aid of PDMS chambers. After cells adhered on the film, the chambers were peeled off. The two sides of the film were coated with two components of the fibrin glue and the film was rolled around an ePTFE mandrel with proper outer diameters by hand. The mandrel was gently extracted. The residual glue components in the scaffolds was washed with PBS (not shown in the figure). Please see also ref. 1 for a more detailed instruction of the fabrication process.

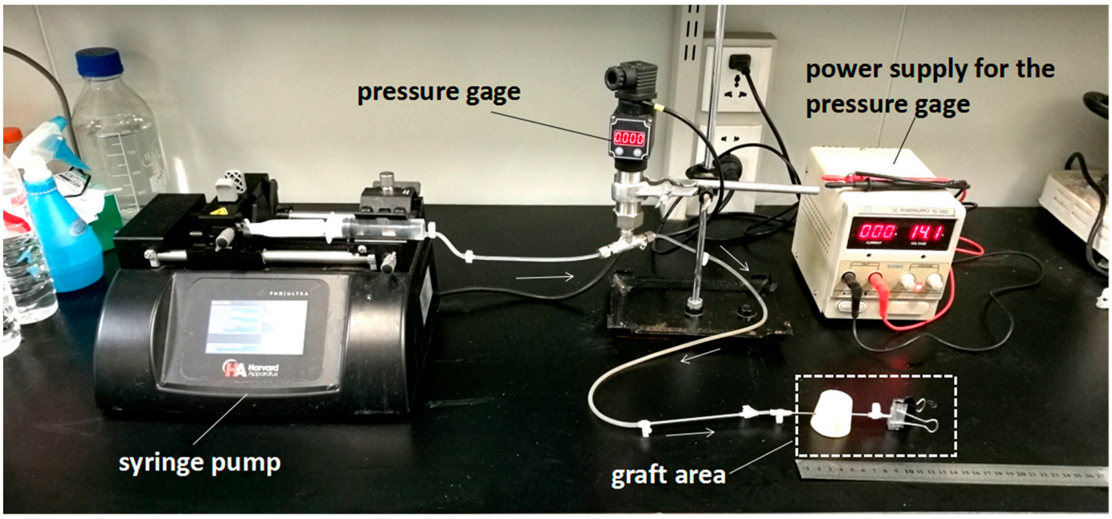

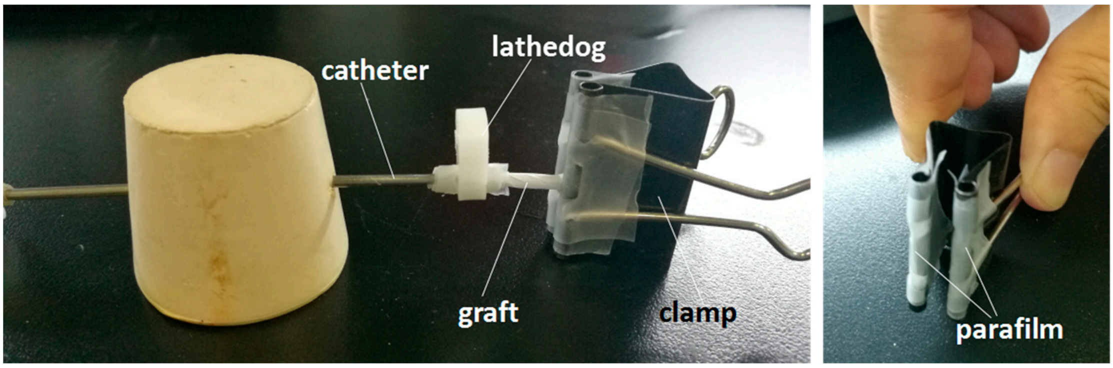

Figure S4. Setup for burst pressure test. The main components of the setup include a syringe pump, a syringe, a pressure gage, and a power supply for the pressure gage. In the graft area, one end of the graft is wrapped by parafilms and hermetically connected to the stainless steel catheter by a lathedog; the other end of the graft was clamped by a steel clamp. The surface of the steel clamp was covered by parafilms to protect the injury of the graft wall from the clamp. All tube connections are strengthened by lathedogs. The arrow heads show the flow direction.

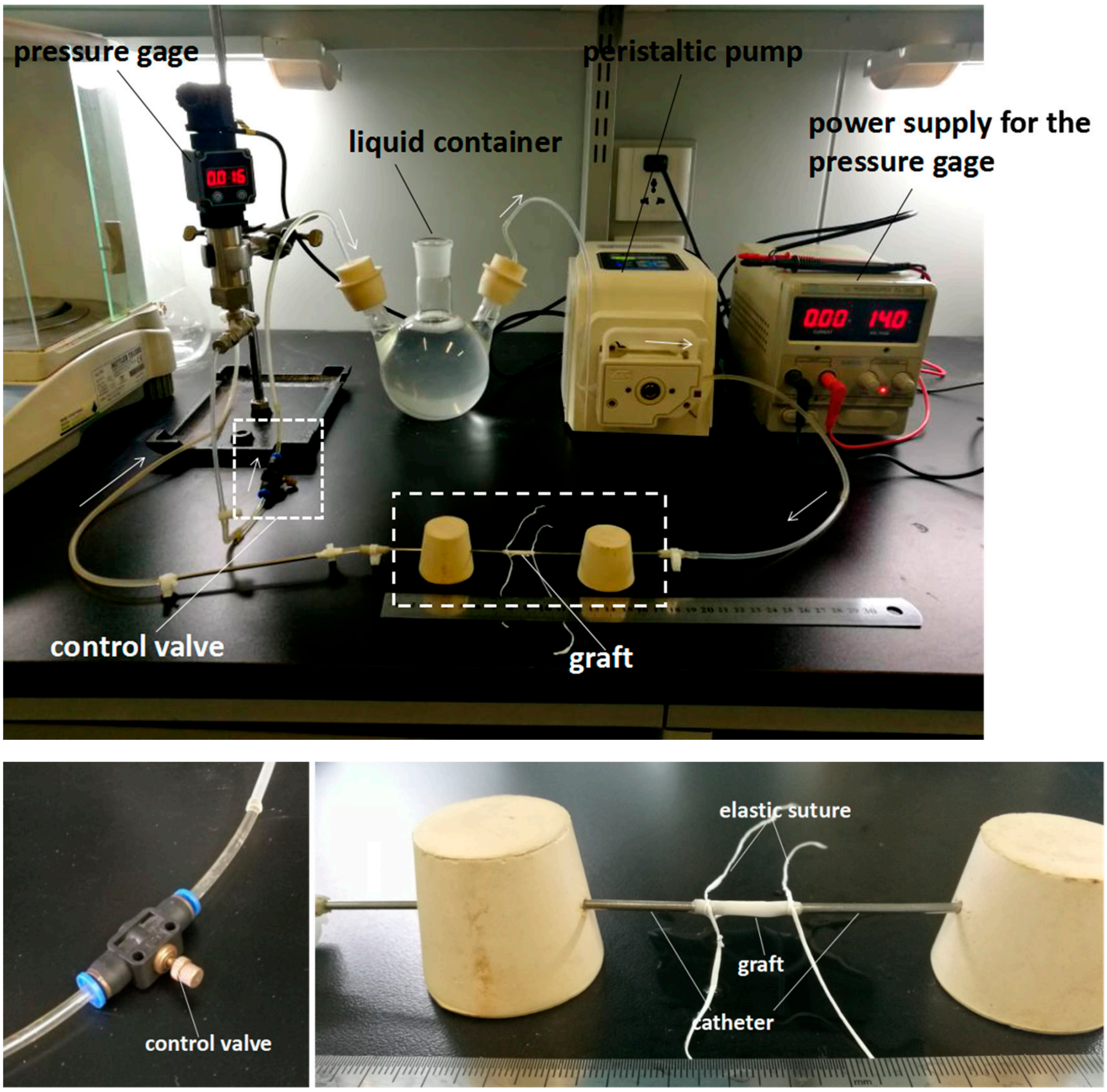

Figure S5. Setup for water leakage and compliance tests. The main components of the setup include a liquid container, a peristaltic pump, a control valve, a pressure gage, and a power supply for the pressure gage. The frequency of the peristaltic pump is set as 75 pulse/min to mimic the heart bite in humans. The control valve is used to control the flow speed and thus the pressure in the conduit. The graft is connected on to the catheters and hermetically sealed with two elastic sutures (teflon film herein). The arrow heads show the flow direction.

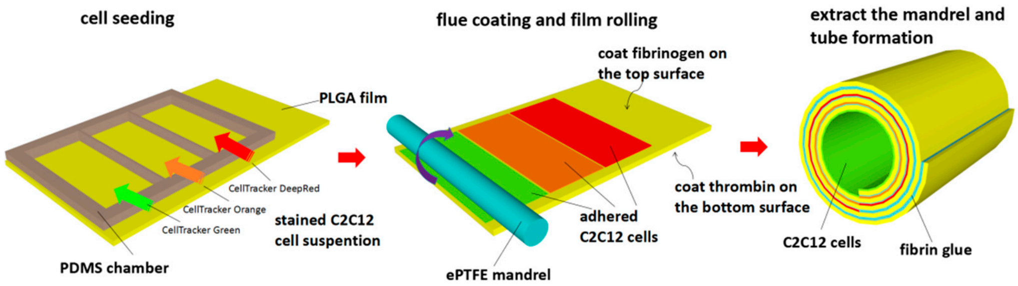

Figure S6. Fabrication scaffolds with stained cells (illustrating the cell distribution for cell migration test by a fluorensent assay). The only difference from the fabrication process for scaffolds without cells is that cells were stained by different dyes before seeding (The cells on the first, second, and third layer were stained with CellTracker Green, CellTracker Orange, and CellTracker DeepRed, respectively).

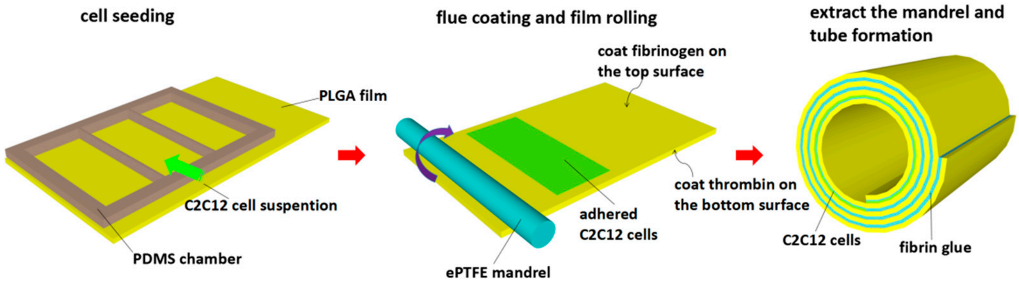

Figure S7. Fabrication scaffolds with one layer of cells seeded (illustrating the cell distribution for cell adhesion, proliferation and migration tests by SEM). The only difference from the fabrication of the scaffolds with three layers is that only the middle part of the chamber is seeded with cell suspension.

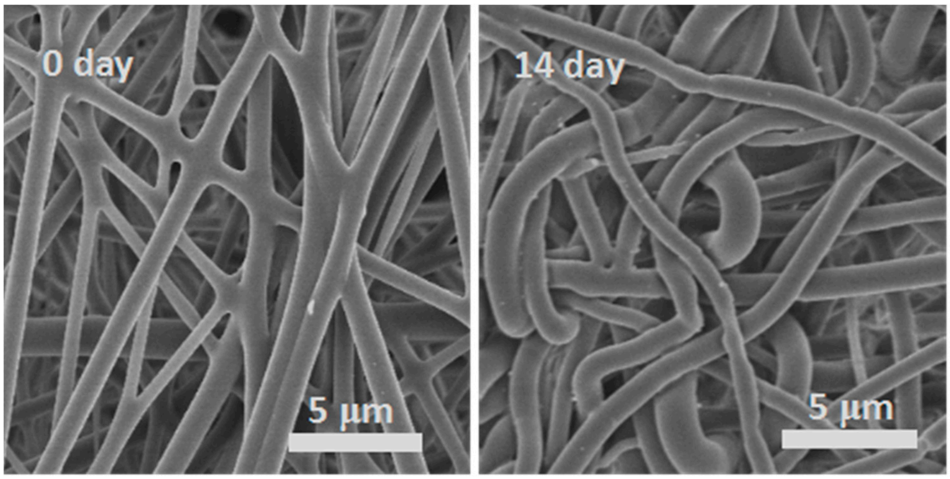

Figure S8. The morphology of PLGA fibers before and after 14 days' degradation in DMEM at 37°C with 5% CO_2.

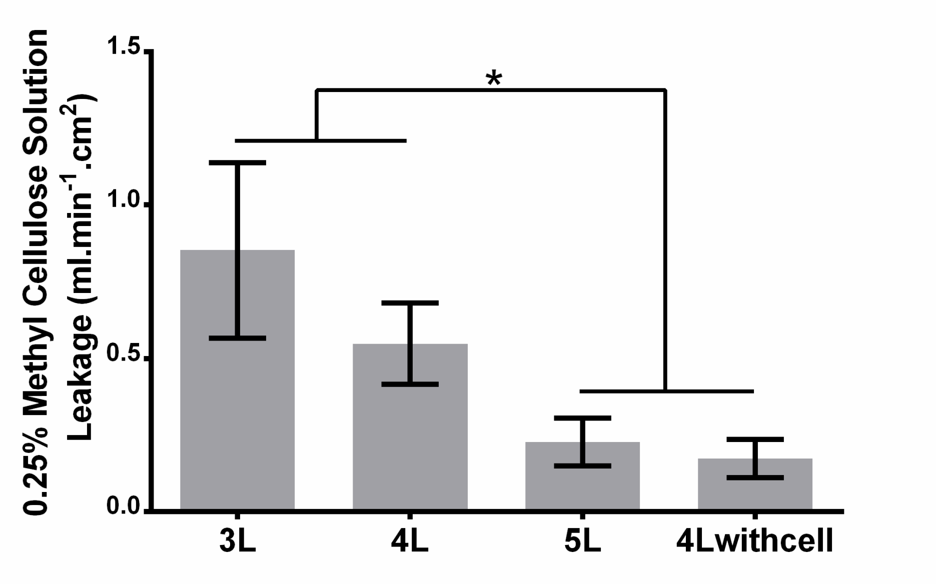

Figure S9. The 0.25% methyl cellulose solution leakage from the scaffolds.

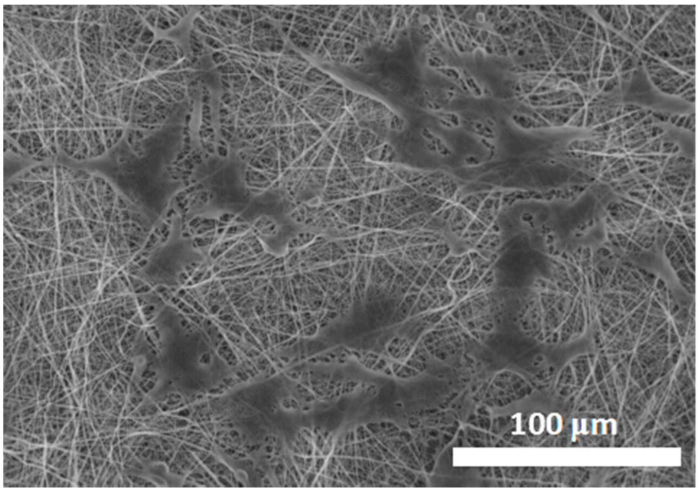

Figure S10. Cell adhesion after 12 h on PLGA ES films.

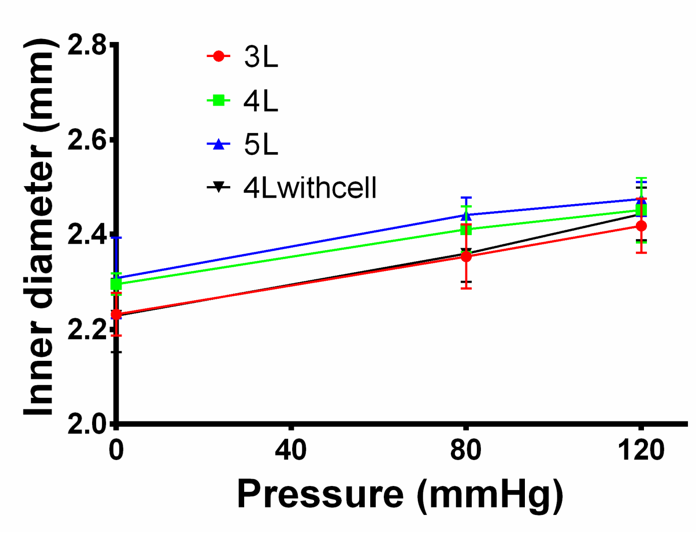

Figure S11. The pressure-inner diameter curve of the scaffolds.

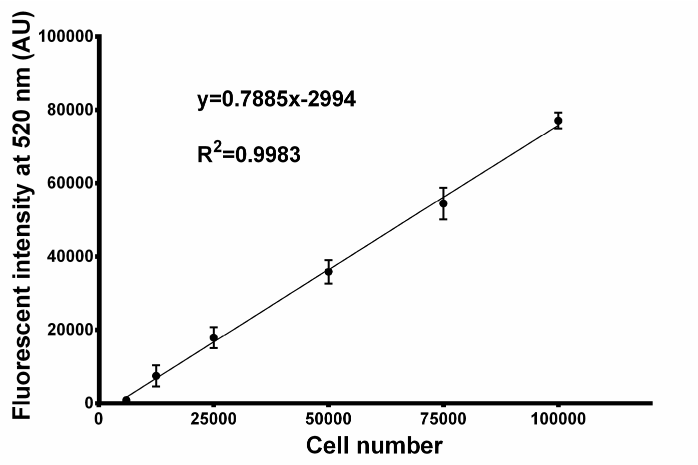

Figure S12. The calibrated standard curve of the CyQuant Cell Proliferation Assay Kit. The standard curve of cell number and fluorescent intensity shows a well linear relationship.

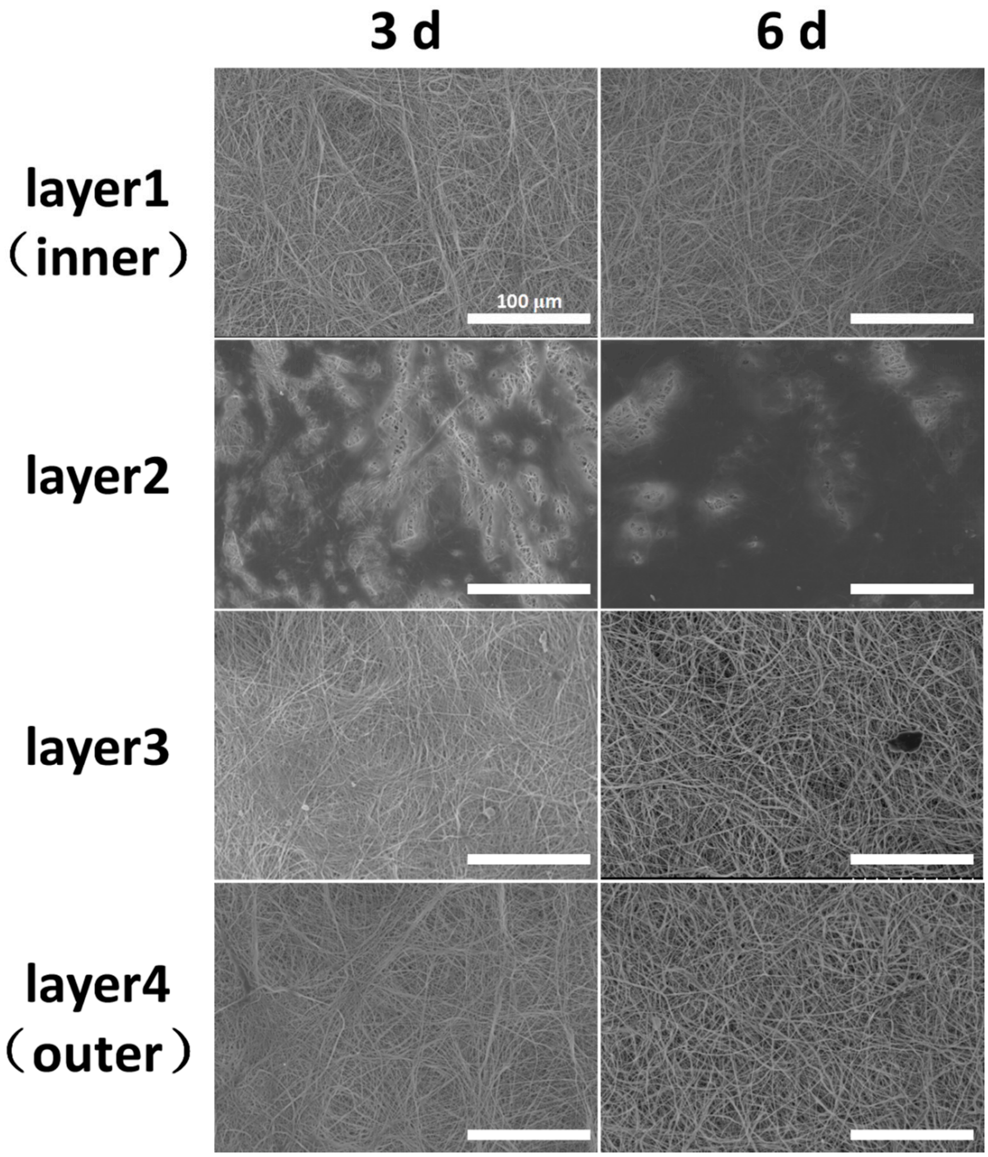

Figure S13. SEM images of and cell proliferation and cell migaration after 3 d and 6 d in scaffolds. The second layer is the cell seeded layer. Cell proliferation can be observed different time points from the second layer. Cell migration between layers can be observed from inner to outer layers of the scaffolds.

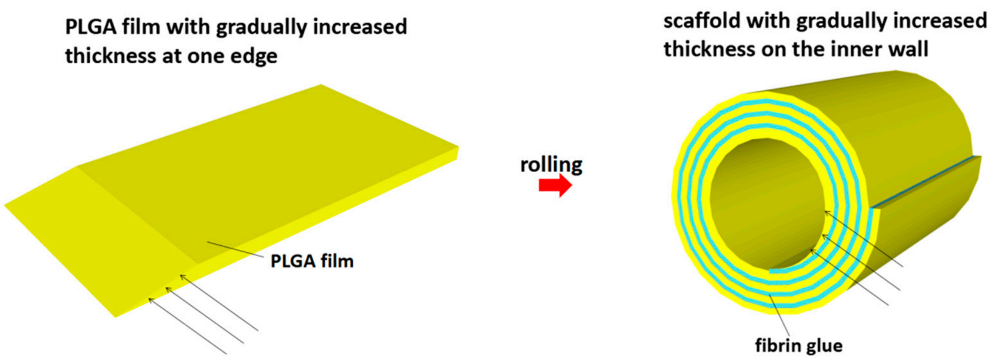

polymers MDPI

Figure S14. A possible design for the solution to the internal edge of the rolling-based scaffolds. A PLGA film with gradually increased thickness at its edge (arrow heads) might be helpful.

Table S1. The relationship between circumferential tensile strain and the Cauthy stress of the scaffolds[&]

	**$T_{\theta\theta}$(P=120 mmHg)**	**$\sigma_{\theta\theta}$(P=120 mmHg)**	**$T_{\theta\theta}$(P=80 mmHg)**	**$\sigma_{\theta\theta}$(P=80 mmHg)**
3L	0.0869±0.0041	155.3±4.2	0.0559±0.011	98.1±3.7
4L	0.0707±0.0388	114.8±7.1	0.0520±0.031	74.0±3.6
5L	0.0757±0.0361	97.9±3.1	0.0603±0.038	63.5±2.3
4Lwithcell	0.1008±0.0246	101.2±3.0	0.0592±0.013	62.0±1.0

[&] $T_{\theta\theta}$ is the circumferential tensile strain and it is dimensionless. $\sigma_{\theta\theta}$ is Cauthy stress and its unit is kPa.

References

1. Wang, N.; Tang, L.; Zheng, W.; Peng, Y.; Cheng, S.; Lei, Y.; Zhang, L.; Hu, B.; Liu, S.; Zhang, W.; Jiang X. Y. A strategy for rapid and facile fabrication of controlled, layered blood vessel-like structures. *RSC A*dvances **2016**, *6*, 55054-55063.
